# Sensitive Fluorescence Assay for the Detection of Alkaline Phosphatase Based on a Cu^2+^-Thiamine System

**DOI:** 10.3390/s21030674

**Published:** 2021-01-20

**Authors:** Han Zhao, Xinfa Liu, Changbei Ma

**Affiliations:** School of Life Sciences, Central South University, Changsha 410013, China; zhaohan202010@163.com (H.Z.); xinfaliu@163.com (X.L.)

**Keywords:** alkaline phosphatase, fluorescence, Cu^2+^-thiamine, inhibitor

## Abstract

The authors describe a novel, facile, and sensitive fluorometric strategy based on a Cu^2+^-thiamine (Cu^2+^-TH) system for the detection of alkaline phosphatase (ALP) activity and inhibition. The principle of the method is as follows. Under a basic conditions, TH, which does not exhibit a fluorescence signal, is oxidized into fluorescent thiochrome (TC) by Cu^2+^. Ascorbic acid 2-phosphate (AAP), which is the enzyme substrate, is hydrolyzed to produce ascorbic acid (AA) by ALP. The newly formed AA then reduces Cu^2+^ to Cu^+^, which prevents the oxidation of TH by Cu^2+^; as a result, the fluorescent signal becomes weaker. On the contrary, in the absence of ALP, AAP cannot reduce Cu^2+^; additions of Cu^2+^ and TH result in a dramatic increase of the fluorescent signal. The sensing strategy displays brilliant sensitivity with a detection limit of 0.08 U/L, and the detection is linear in the concentration range of 0.1 to 100 U/L. This approach was successfully applied to ALP activity in human serum samples, indicating that it is reliable and may be applied to the clinical diagnosis of ALP-related diseases.

## 1. Introduction

Alkaline phosphatase (ALP), an enzyme broadly found in mammalian tissues, can catalyze the hydrolysis and dephosphorylation processes of multifarious substrates, which include nucleic acids, proteins, and some small molecules [1,2,3]. Furthermore, research suggests that ALP is involved in cell regulation and signal transduction processes [4]. The aberrant level of ALP in serum is tightly associated with numerous serious illnesses, such as hepatitis, breast and prostatic carcinoma, liver dysfunction, osteopathy, and diabetes, and it is generally recognized as a biomarker in early clinical diagnosis [5,6,7]. Consequently, attention has been paid to ALP for its potential use in fast and sensitive detection.

Up to now, a variety of analytical techniques, including electrochemistry, fluorometry, colorimetry, chromatography, and surface-enhanced Raman scattering have been used in the determination of ALP activity [8,9,10,11,12,13,14,15,16,17]. For example, Wu et al. established a signal amplified electrochemical method which relied on enzyme-induced metallization (EIM) for ALP analysis [18]. Despite its low detection limit, this method has other disadvantages: the preparation of modified electrodes and the sample preprocessing procedures are costly, complicated, and time-consuming. Tang et al. employed a colorimetric sensing platform for ALP activity assay by the use of Cu(II)-modulated G-quadruplex-based DNAzymes [19]. This colorimetric method has some advantages, such as simple instruments and convenient operation, however, the method is inefficient and uses probes with a complicated design. Recently, increasing attention has been paid to fluorescence-based strategies for their rapid reaction time and considerably high sensitivity. The fluorescence probes used this strategy are mainly composed of organic dyes, quantum dots (QDs), DNA-templated nanoparticles, and conjugated polyelectrolytes [20,21,22,23]. For this purpose, Qu et al. proposed a turn-on fluorescence method based on carbon dots and MnO_2_ nanosheets for the detection of ALP [24]. He et al. developed a label-free fluorometric approach for the detection of ALP through the synthesis of ssDNA-templated fluorescent silver nanoclusters [25]. However, these strategies have unavoidable disadvantages, such as the fact that QDs have high toxicity, dye-labeling steps are complex, the synthetic process is long, and the cost of reagents is high. Hence, the development of a facile, economical, and timesaving assay of ALP is highly desirable.

Thiamine (TH), also known as vitamin B1, has been utilized as a non-fluorescent substrate [26,27,28]. It has attracted tremendous attention for its low cost, good water-solubility, and easy accessibility. TH does not exhibit a fluorescence signal; however, under basic conditions, it can be easily converted into fluorescent thiochrome (TC) by Cu^2+^ [29]. Thus, the development of a Cu^2+^-TH-based system may open up a new avenue for fluorescence detection. In this study, we propose a novel Cu^2+^-TH-based fluorometric strategy system for the detection of ALP activity. The addition of ALP can catalyze the hydrolysis of ascorbic acid 2-phosphate (AAP) into ascorbic acid (AA), which can then reduce Cu^2+^ to Cu^+^ that in turn leads to fluorescence quenching. In the absence of ALP, Cu^2+^ can oxidize TH and convert it into TC, recovering the fluorescence signal. The present fluorescent method, which has high selectivity and sensitivity, was triumphantly utilized to detect ALP in human serum samples. It is a promising strategy that can be employed in various clinical applications.

## 2. Materials and Methods

### 2.1. Materials and Reagents

Alkaline phosphatase (ALP), uracil DNA glycosylase (UDG), T4 polynucleotide kinase (T4 PNK), and 8-hydroxy guanine DNA glycosidase (hOGG1) were all acquired from Takara Biotechnology Co., Ltd. (Dalian, China). Ascorbic acid 2-phosphate (AAP), glycine (Gly), alanine (Ala), serine (Ser), glutamic acid (Glu), histidine (His), lysozyme, streptavidin (SA), glucose (C_6_H_12_O_6_), thiamine, Na_3_VO_4_, and ATP were purchased from Source Leaf Biotechnology Co., Ltd. (Shanghai, China). Cupric sulfate (CuSO_4_), sodium hydroxide (NaOH), and 3′-(N-morpholino) propanesulfonic acid (MOPS) were purchased from Sinopharm Chemical Reagent Co., Ltd. (Shanghai, China). All other chemicals were of analytical grades. All of the solutions used in this study were prepared with ultrapure water (18.2 MΩ.cm) obtained from a Milli-Q water purification system.

### 2.2. Apparatus

Fluorescence measurements were conducted at room temperature on an F-2700 fluorescence spectrophotometer (Hitachi, Japan). The fluorescence emission spectra were recorded in the range of 400–570 nm with the excitation wavelength set at 370 nm. The slit widths for excitation and emission were both set at 10 nm.

### 2.3. Optimization of ALP Detection

To acquire the optimal experimental performance, a variety of reaction conditions were optimized including the concentration of Cu^2+^, AAP, TH, and reaction time between ALP and AAP. The concentration range of Cu^2+^, AAP, and TH were 2–50 μM, 200–800 μM, and 20–400 μM, respectively. The range of reaction time between ALP, AAP, and the Cu^2+^-TH system was from 5 min to 45 min.

### 2.4. Fluorescence-Based Determination of ALP Activity

Quantitative determination of ALP activity based on the Cu(II)-TH system was carried out as follows: 500 μM AAP was incubated with different ALP activities ranging from 0 to 1500 U/L in a MOPS buffer (10 mM, pH 8.0). The mixture was incubated at 37 °C for 30 min, and then Cu^2+^ (20 μM) was added to the above-mentioned solution at room temperature for 25 min. Subsequently, TH (100 μM) and NaOH (50 mM) were added to a final volume of 100 μL. The mixture was vortexed and reacted for 15 min at 25 °C.

### 2.5. Selectivity of ALP

To assess the selectivity of the proposed method, a variety of common molecules, including UDG, hOGG1, T4 PNK, SA, lysozyme, Gly, Ala, Ser, Glu, His, C_6_H_12_O_6_, and ATP, were used as interferences for comparison. First, different molecules and 500 μM AAP were respectively mixed with the reaction buffer (10 mM MOPS buffer, pH 8.0) at 37 °C for 30 min. Afterward, Cu^2+^ (20 μM) was incubated with the above-prepared solution at 25 °C for 25 min. Finally, 100 μM TH and 50 mM NaOH were added into the mixture at 25 °C for 15 min prior to fluorescence measurement.

### 2.6. Inhibition Investigation

To examine the effect of Na_3_VO_4_ inhibition on ALP activity, a series of experiments were carried out. First, various concentrations of Na_3_VO_4_ (0, 100, 300, 500, 600, 800, and 1000 μM) were mixed with ALP (100 U/L) and AAP (500 μM) in an MOPS buffer (10 mM, pH 8.0) and incubated at 37 °C for 30 min. Then, Cu^2+^ (20 μM) was added, and the reaction solutions were allowed to incubate at ambient temperature for 25 min. Finally, TH (100 μM) and NaOH (50 mM) were added. The relative activity was obtained according to the following equation:

$$\mathrm{Relative}\;\mathrm{activity}\;(\%)\;=\;\frac{(F_{\left(\mathrm{inhibitor}\right)}-F_{0})}{(F_{\left(\mathrm{no}\;\mathrm{inhibitor}\right)}-F_{0})}\times 100$$

where F (inhibitor) stands for the fluorescence intensity of the samples in the presence of ALP and the inhibitor, F (no inhibitor) stands for the fluorescence intensity of the samples with ALP but no inhibitor, F_0_ stands for the fluorescence intensity without ALP or the inhibitor.

### 2.7. ALP Activity Assay in Human Serum Samples

To evaluate the feasibility of the proposed strategy in detecting ALP in real samples, a certain amount of ALP was spiked into human serum samples, which were diluted by 100 times with a reaction buffer (10 mM MOPS, pH 8.0). The ALP activity of the sample was subsequently determined by fluorescent measurement.

### 2.8. Statistical Analysis

Data analysis was performed with GraphPad Prism 8.0 statistical software. The results between two groups were analyzed using two-tailed Student’s t-tests. *p* < 0.05 was considered to indicate a significant difference.

## 3. Results and Discussion

### 3.1. Principles of ALP Activity Detection

**Scheme 1:**
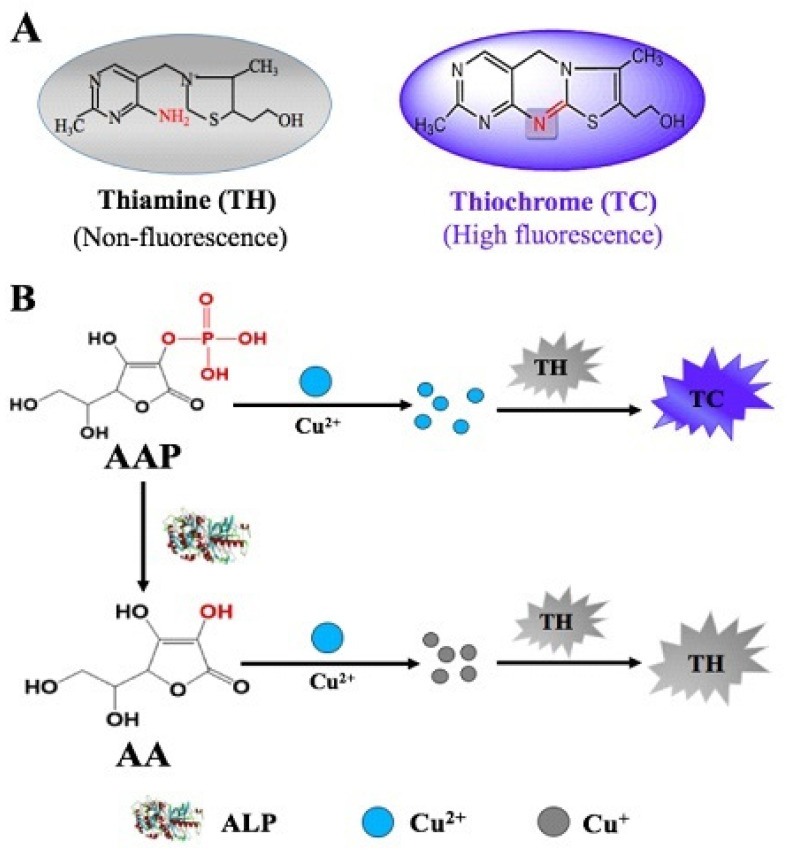
(**A**) The structures of thiamine (TH) and thiochrome (TC). (**B**) Schematic illustration showing the principle of ALP activity assay based on a Cu^2+^-TH system.

The illustration for the principle of the proposed fluorescence-based Cu^2+^-TH system in the determination of ALP activity is depicted in Scheme 1. In a basic solution, TH can be oxidized by Cu^2+^ to produce fluorescent thiochrome (TC). In the absence of ALP, the fluorescence signal is observed in the reaction system consisting of AAP, Cu^2+^, and TH. In the presence of ALP, AAP, which acts as the substrate for ALP dephosphorylation, is hydrolyzed to generate ascorbic acid. In the presence of ascorbic acid, which can reduce metal ions, Cu^2+^ is reduce to Cu^+^. As a result, the oxidation of TH is impaired, causing reduced fluorescence intensity. Variation of the fluorescence response with the ALP concentration indicates that a timesaving, facile fluorescent method for ALP detection is successfully developed.

### 3.2. Validation of ALP Assay

**Figure 1:**
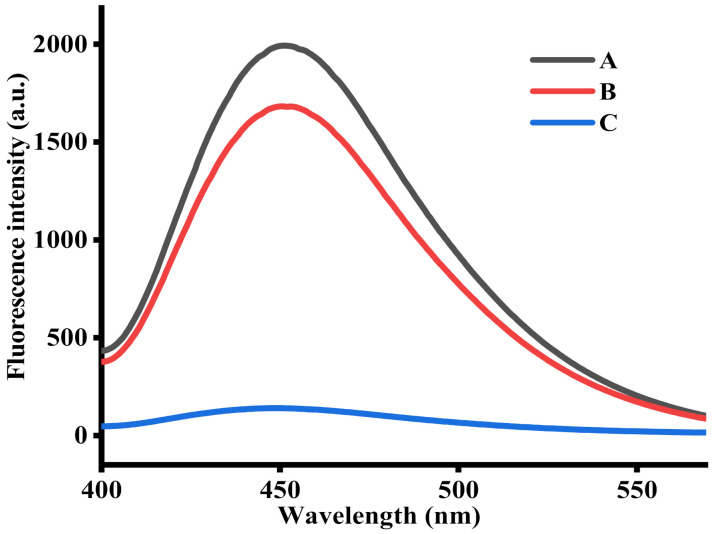
Fluorescence emission spectra obtained under different conditions: (**A**) Cu^2+^ + TH; (**B**) AAP + Cu^2+^ + TH; and (**C**) ALP + AAP + Cu^2+^ + TH. The concentrations of ALP, AAP, Cu^2+^, and TH were 1000 U/L, 500 μM, 20 μM, and 100 μM, respectively.

To examine the feasibility of this method, a series of tests were performed. As shown in Figure 1, a mixture containing Cu^2+^, TH, and NaOH exhibited a remarkable fluorescence signal (curve A), suggesting the TC was generated from the oxidation of TH by Cu^2+^. After adding AAP to the reaction system, the fluorescence intensity decreased a little, indicating that AAP has a certain influence on the fluorescence signal of TC, but its influence on the overall detection performance of the sensor platform is relatively low. By contrast, as indicated by curve C, the fluorescence intensity sharply decreased after the hydrolysis of AAP by ALP. This indicates that Cu^2+^ was reduced by ascorbic acid, thereby the oxidation of TH was disrupted. Based on the above, a convenient, sensitive fluorescent assay for ALP activity was achieved.

### 3.3. Optimization of Experimental Conditions

**Figure 2:**
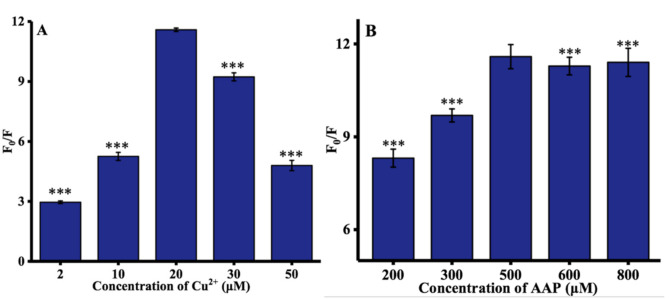

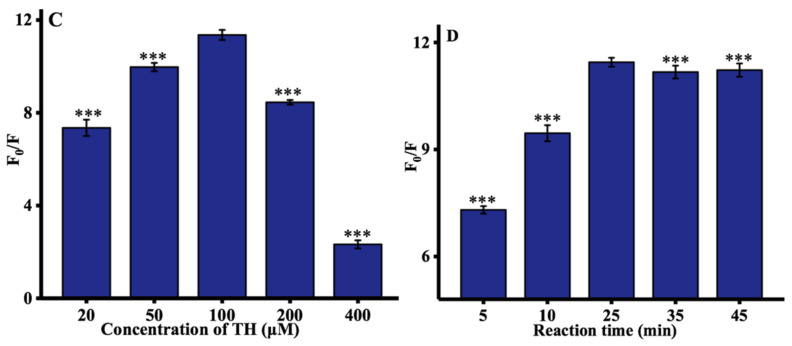
Optimization of experimental conditions: (**A**) concentration of Cu^2+^; (**B**) concentration of AAP; (**C**) concentration of TH; and (**D**) reaction time. F0 and F represented the fluorescence intensities in the absence and presence of ALP. Error bars were estimated from three replicate measurements. *** *p* < 0.001 compared with optimal concentration, unpaired *t* test.

Some experimental parameters were further optimized to improve the performance of ALP assay, including the concentrations of Cu^2+^, AAP, and TH, and time at which Cu^2+^ is reduced by ascorbic acid. The concentration of Cu^2+^ (from 2 to 50 μM) was first investigated. As depicted in Figure 2A, the optimal concentration of Cu^2+^ was 20 μM. The concentration of the AAP was then optimized. As illustrated in Figure 2B, with increasing the AAP concentration, the hydrolysis of AAP by ALP was more complete, thereby resulting in a significant increase of the ratio of fluorescence intensity at 445 nm until this reached a plateau at 500 μM. Moreover, the influence of TH concentration on the fluorescence spectra of TC was determined, and 100 μM TH was used throughout the experiments, as shown in Figure 2C. Finally, as can be observed in Figure 2D, the rate of fluorescence significantly increased with increasing reduction time until reaching a maximum value after 25 min. Hence, 25 min was chosen as the optimized ALP reaction time.

### 3.4. Determination of ALP Activity by the Proposed Method

**Figure 3:**
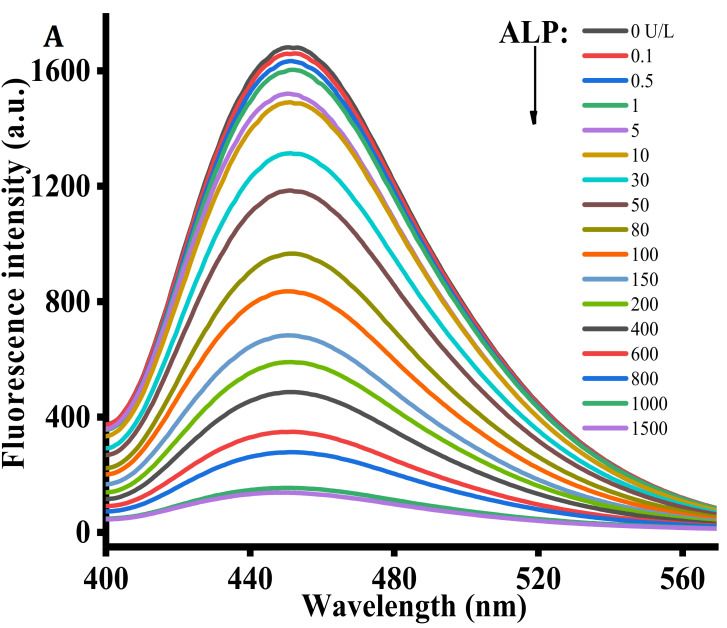

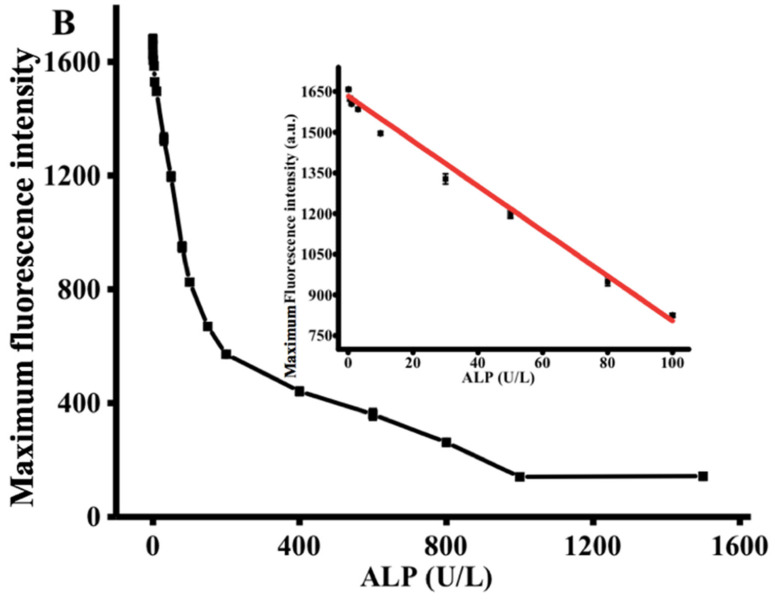
(**A**) Fluorescence emission spectra obtained using different concentrations of ALP (0, 0.1, 0.5, 1, 5, 10, 30, 50, 80, 100, 150, 200, 400, 600, 800, 1000, and 1500 U/L). (**B**) The relationship between the fluorescence intensity at 445 nm and various ALP concentrations. AAP = 500 μM, Cu^2+^ = 20 μM, TH = 100 μM, ALP reaction time = 30 min, reaction time between the Cu^2+^-TH system and ALP, AAP = 25 min. Error bars were estimated from three replicate measurements.

Under the optimized conditions, the performance of the proposed strategy in detecting ALP activity was examined by comparing the ALP activity of the reactions containing different ALP concentrations. Figure 3A exhibits the diverse fluorescent signal response in the presence of different ALP levels. With increasing ALP concentration from 0 to 1500 U/L, the fluorescence intensity at 445 nm gradually decreased, reaching a plateau at an ALP concentration of 1000 U/L. As shown in the inset of Figure 3B, a linear correlation between fluorescence intensity and ALP concentrations is observed at the concentration region of 0.1 to 100 U/L. The corresponding regression equation is F = −8.068 C + 1605.267 (R^2^ = 0.99), in which F represents the peak intensity and C is the concentration of ALP (U/L). The limit of detection for ALP was estimated to be 0.08 U/L according to 3σ rule. In comparison with the previously reported ALP detection method (Table 1), the proposed method has lower detection limit and is more cost-effective [30,31,32].

### 3.5. Selectivity for ALP Activity Assay

**Figure 4:**
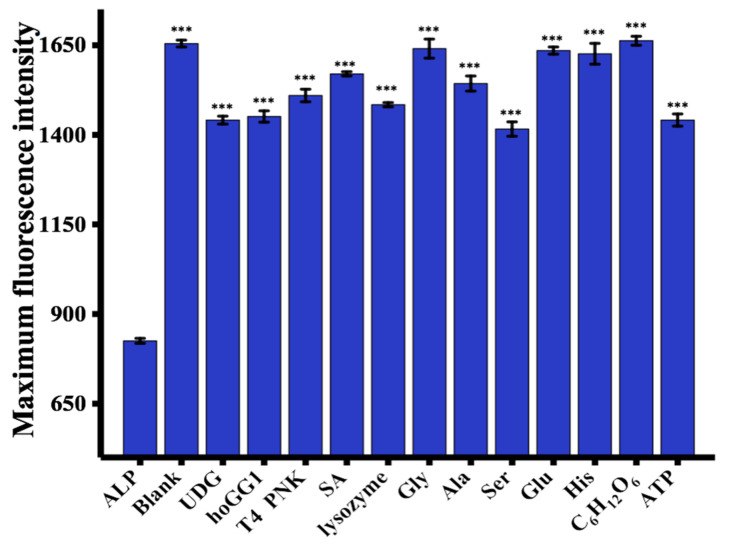
Sensitivity of the proposed assay. The fluorescence intensity was measured at 445 nm. ALP concentration = 100 U/L. UDG, hOGG1, and T4 PNK concentrations = 1000 U/L. SA, lysozyme, Gly, Ala, Ser, Glu, His, C_6_H_12_O_6_, and ATP concentrations = 0.5 μM. *** *p* < 0.001 compared with ALP.

To determine the selectivity of the proposed strategy, the fluorescence signal in response to a variety of molecules, including UDG, hOGG1, T4 PNK, SA, lysozyme, Gly, Ala, Ser, Glu, His, C_6_H_12_O_6_, and ATP was measured [19]. As illustrated in Figure 4, a negligible fluorescence response was observed in the presence of ALP compared with the blank control and other proteins and potential interferents, which suggests the good selectivity of this strategy for ALP assay.

### 3.6. Assay of ALP Activity Inhibitor

**Figure 5:**
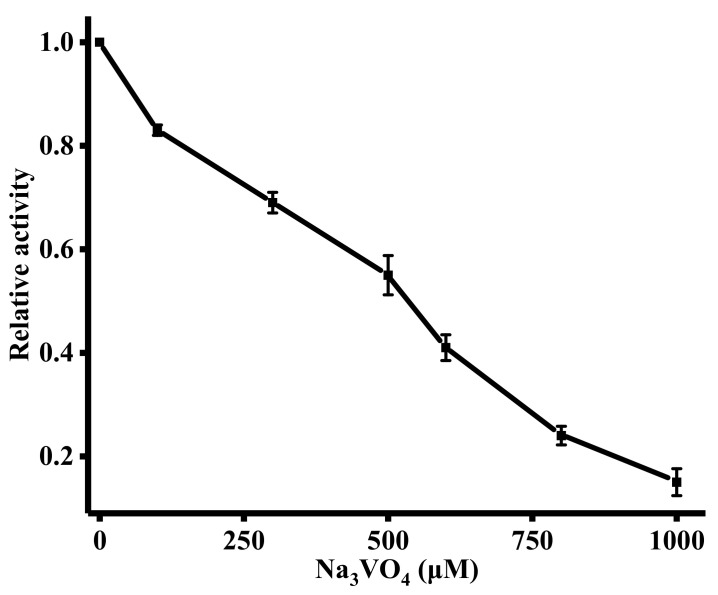
(A) Influence of Na_3_VO_4_ (0, 100, 300, 500, 600, 800, and 1000 μM) on the activity of ALP. The concentration of ALP was 1000 U/L. Error bars were estimated from three replicate measurements.

The ability of this sensing strategy to evaluate the influence of the ALP inhibitor on ALP activity is of great significance because it can be applied to drug screening and disease therapy. A known the ALP inhibitor, Na_3_VO_4_, was employed in the inhibitory assays [33]. In Figure 5, it was observed that ALP activity was gradually inhibited with the increasing concentration of Na_3_VO_4_. The IC50 value (half-maximal inhibitory concentration) of Na_3_VO_4_ was 262 μM. The results suggest that our method is suitable for screening of the ALP inhibitor.

### 3.7. Analysis of ALP in Human Serum Samples

Practical use of this proposed approach was examined by spiked recovery experiments using 1% human serum. As seen in Table 2, the recovery rates of 101.3%, 97.06%, and 102.9% were obtained from the samples spiked with 10, 50, and 80 U/L of ALP, respectively, suggesting that our method is promising and feasible for ALP activity assay in biological samples.

## 4. Conclusions

In summary, a label-free, sensitive, and time-saving Cu^2+^-TH-system-fluorometric method for ALP activity determination was successfully constructed. This strategy relies on the ability of AA, which is derived from the hydrolysis of AAP by ALP, to reduce Cu^2+^ to Cu^+^. The oxidation of TH by Cu^2+^ is prevented causing the fluorescent signal to reduce. The detection limit of the proposed strategy was 0.08 U/L, which is much lower than that of various other strategies reported in the literature. Other benefits of the Cu^2+^-TH system include high sensitivity, low cost, and facile operation, and it also does not require fluorescent or nanomaterial probes. Additionally, the strategy was successfully used to assay ALP activity in real samples, and satisfactory results were achieved. This further indicates that this approach can potentially provide a platform for the diagnosis of ALP-related diseases.

## Figures and Tables

**Table 1:** Comparison of different methods for ALP activity detection.

Method	Material	LOD (U/L)	Dynamic Range (U/L)	Reference
Colorimetry	Cu^2+^-HRP-TMB-H_2_O_2_ system	5.4	0–120	15
Colorimetry	Cu-MOFs	0.19	1–34	14
Electrochemistry	AuNPs/glassy carbon	0.03	0.1–5	9
Fluorescence	Cu(II)-dependent DNAzyme	0.14	0.36–54.55	12
Fluorescence	Thioflavin T	1	1–200	32
Fluorescence	AuNPs and nanorod	0.06	0.2–100	33
Fluorescence	Carbon quantum dot	1.1	6.7–782.6	34
Fluorescence	Cu^2+^-TH system	0.08	0.1–100	This work

**Table 2:** Recovery experiments of ALP in human serum samples.

Sample	Added (U/L)	Found (U/L)	Recovery (%)
1	10	10.13 ± 0.78	101.3
2	50	48.53 ± 0.24	97.06
3	80	82.34 ± 0.69	102.9

## Data Availability

The data presented in this study are available on request from the corresponding author. The data are not publicly available due to participant confidentiality.

## References

[1] Coleman J.E. (1992). Structure and mechanism of alkaline phosphatase. Annu. Rev. Biophys. Biomol. Struct..

[2] Harris H. (1990). The human alkaline phosphatases: What we know and what we don’t know. Clin. Chim. Acta.

[3] Tang Z., Chen H., He H., Ma C. (2019). Assays for alkaline phosphatase activity: Progress and prospects. TrAC Trends Anal. Chem..

[4] Julien S.G., Dubé N., Hardy S., Tremblay M.L. (2011). Inside the human cancer tyrosine phosphatome. Nat. Rev. Cancer.

[5] Ooi K., Shiraki K., Morishita Y., Nobori T. (2007). High-molecular intestinal alkaline phosphatase in chronic liver diseases. J. Clin. Lab. Anal..

[6] Colombatto P., Randone A., Civitico G., Gorin J.M., Dolci L., Medaina N., Oliveri F., Verme G., Marchiaro G., Pagni R. (1996). Hepatitis G virus RNA in the serum of patients with elevated gamma glutamyl transpeptidase and alkaline phosphatase: A specific liver disease. J. Viral Hepat..

[7] Lorente J.A., Valenzuela H., Morote J., Gelabert A. (1999). Serum bone alkaline phosphatase levels enhance the clinical utility of prostate specific antigen in the staging of newly diagnosed prostate cancer patients. Eur. J. Nucl. Med. Mol. Imaging.

[8] Li X., Zhu L., Zhou Y., Yin H., Ai S. (2017). Enhanced Photoelectrochemical Method for Sensitive Detection of Protein Kinase a Activity Using TiO_2_/g-C_3_N_4_, PAMAM Dendrimer, and Alkaline Phosphatase. Anal. Chem..

[9] Liu Y.Q., Xiong E.H., Li X.Y., Li J.J., Zhang X.H., Chen J.H. (2017). Sensitive electrochemical assay of alkaline phosphatase activity based on TdT-mediated hemin/G-quadruplex DNAzyme nanowires for signal amplification. Biosens. Bioelectron..

[10] Ma J.-L., Yin B.-C., Wu X., Ye B.-C. (2016). Copper-Mediated DNA-Scaffolded Silver Nanocluster On–Off Switch for Detection of Pyrophosphate and Alkaline Phosphatase. Anal. Chem..

[11] Liu H.S., Ma C.B., Wang J., Wang K.M., Wu K.F. (2017). A turn-on fluorescent method for determination of the activity of alkaline phosphatase based on dsDNA-templated copper nanoparticles and exonuclease based amplification. Microchim. Acta.

[12] Zhao M., Guo Y., Wang L., Luo F., Lin C., Lin Z., Chen G. (2016). A sensitive fluorescence biosensor for alkaline phosphatase activity based on the Cu(II)-dependent DNAzyme. Anal. Chim. Acta.

[13] Mei Y., Hu Q., Zhou B., Zhang Y., He M., Xu T., Li F., Kong J. (2018). Fluorescence quenching based alkaline phosphatase activity detection. Talanta.

[14] Wang C., Gao J., Cao Y., Tan H. (2018). Colorimetric logic gate for alkaline phosphatase based on copper (II)-based metal-organic frameworks with peroxidase-like activity. Anal. Chim. Acta.

[15] Zhang X., Sun Y., Lin L., Shi C., Wang G., Zhang X. (2016). Naked-eye sensitive detection of alkaline phosphatase (ALP) and pyrophosphate (PPi) based on a horseradish peroxidase catalytic colorimetric system with Cu(ii). Analyst.

[16] Lakra S., Jadhav V.J., Garg S.R. (2016). Development of a Chromatographic Method for the Determination of Alkaline Phosphatase Activity in Pasteurized Milk. Food Anal. Methods.

[17] Ruan C., Wang W., Gu B. (2006). Detection of Alkaline Phosphatase Using Surface-Enhanced Raman Spectroscopy. Anal. Chem..

[18] Wu Z., Zhou C.-H., Pan L.-J., Zeng T., Zhu L., Pang D.-W., Zhang Z.-L. (2016). Reliable Digital Single Molecule Electrochemistry for Ultrasensitive Alkaline Phosphatase Detection. Anal. Chem..

[19] Tang Z., Zhang H., Ma C., Gu P., Zhang G., Wu K., Chen M., Wang K. (2018). Colorimetric determination of the activity of alkaline phosphatase based on the use of Cu(II)-modulated G-quadruplex-based DNAzymes. Microchim. Acta.

[20] Dong L., Miao Q., Hai Z., Yuan Y., Liang G. (2015). Enzymatic Hydrogelation-Induced Fluorescence Turn-Off for Sensing Alkaline Phosphatase in Vitro and in Living Cells. Anal. Chem..

[21] Guo L., Chen D., Yang M. (2017). DNA-templated silver nanoclusters for fluorometric determination of the activity and inhibition of alkaline phosphatase. Microchim. Acta.

[22] Liu Y., Schanze K.S. (2008). Conjugated Polyelectrolyte-Based Real-Time Fluorescence Assay for Alkaline Phosphatase with Pyrophosphate as Substrate. Anal. Chem..

[23] Qian Z., Chai L., Tang C., Huang Y., Chen J., Feng H. (2015). Carbon Quantum Dots-Based Recyclable Real-Time Fluorescence Assay for Alkaline Phosphatase with Adenosine Triphosphate as Substrate. Anal. Chem..

[24] Qu F., Pei H., Kong R.-M., Zhu S., Xia L. (2017). Novel turn-on fluorescent detection of alkaline phosphatase based on green synthesized carbon dots and MnO_2_ nanosheets. Talanta.

[25] He Y., Jiao B.N. (2017). Determination of the activity of alkaline phosphatase based on the use of ssDNA-templated fluorescent silver nanoclusters and on enzyme-triggered silver reduction. Microchim. Acta.

[26] Tan H., Li Q., Zhou Z., Ma C., Song Y., Xu F., Wang L. (2015). A sensitive fluorescent assay for thiamine based on metal-organic frameworks with intrinsic peroxidase-like activity. Anal. Chim. Acta.

[27] Ni P., Chen C., Jiang Y., Zhao Z., Lu Y. (2018). Fluorometric determination of sulfide ions via its inhibitory effect on the oxidation of thiamine by Cu(II) ions. Microchim. Acta.

[28] Purbia R., Paria S. (2016). A simple turn on fluorescent sensor for the selective detection of thiamine using coconut water derived luminescent carbon dots. Biosens. Bioelectron..

[29] Perezruiz T., Martinezlozano C., Tomas V., Ibarra I. (1992). Flow injection fluorimetric determination of thiamine and copper based on the formation of thiochrome. Talanta.

[30] Du J., Xiong L., Ma C., Liu H., Wang J., Wang K. (2016). Label-free DNA hairpin probe for real-time monitoring of alkaline phosphatase activity. Anal. Methods.

[31] Xu A.-Z., Zhang L., Zeng H.-H., Liang R.-P., Qiu J.-D. (2018). Fluorometric determination of the activity of alkaline phosphatase based on the competitive binding of gold nanoparticles and pyrophosphate to CePO_4_:Tb nanorods. Microchim. Acta.

[32] Qian Z.S., Chai L.J., Huang Y.Y., Tang C., Shen J.J., Chen J.R., Feng H. (2015). A real-time fluorescent assay for the detection of alkaline phosphatase activity based on carbon quantum dots. Biosens. Bioelectron..

[33] Gibbons I.R., Cosson M.P., Evans J.A., Gibbons B.H., Houck B., Martinson K.H., Sale W.S., Tang W.J. (1978). Potent inhibition of dynein adenosinetriphosphatase and of the motility of cilia and sperm flagella by vanadate. Proc. Natl. Acad. Sci. USA.

